# Mapping Language Networks Using the Structural and Dynamic Brain Connectomes

**DOI:** 10.1523/ENEURO.0204-17.2017

**Published:** 2017-11-06

**Authors:** John Del Gaizo, Julius Fridriksson, Grigori Yourganov, Argye E. Hillis, Gregory Hickok, Bratislav Misic, Chris Rorden, Leonardo Bonilha

**Affiliations:** 1 Department of Neurology, Medical University of South Carolina, Charleston, SC 29425; 2 Department of Communication Sciences and Disorders, University of South Carolina, Columbia, SC 29208; 3 Department of Neurology, Johns Hopkins University, Baltimore, MD 21218; 4 Department of Cognitive Sciences, University of California Irvine, Irvine, CA 92697; 5 Montreal Neurological Institute, McGill University, QC, H3A 0G4, Canada; 6 Department of Psychology, University of South Carolina, Columbia, SC 29208

**Keywords:** aphasia, connectome, diffusion tensor imaging, stroke

## Abstract

Lesion-symptom mapping is often employed to define brain structures that are crucial for human behavior. Even though poststroke deficits result from gray matter damage as well as secondary white matter loss, the impact of structural disconnection is overlooked by conventional lesion-symptom mapping because it does not measure loss of connectivity beyond the stroke lesion. This study describes how traditional lesion mapping can be combined with structural connectome lesion symptom mapping (CLSM) and connectome dynamics lesion symptom mapping (CDLSM) to relate residual white matter networks to behavior. Using data from a large cohort of stroke survivors with aphasia, we observed improved prediction of aphasia severity when traditional lesion symptom mapping was combined with CLSM and CDLSM. Moreover, only CLSM and CDLSM disclosed the importance of temporal-parietal junction connections in aphasia severity. In summary, connectome measures can uniquely reveal brain networks that are necessary for function, improving the traditional lesion symptom mapping approach.

## Significance Statement

In this article, we present a description of how the individualized structural connectome can be measured from individuals with large brain lesions, leveraging recently described approaches to calculate network dynamics. We then tested whether structural and dynamic connectome measures could be combined with conventional lesion-symptom mapping to better predict and elucidate the neuroanatomy of poststroke aphasia. Using machine learning applied to a large dataset of stroke survivors, we not only observed that adding connectome measures to lesion mapping improves the prediction of aphasia severity, but connectome measures disclose crucial language networks, notably within the temporal parietal regions, that are not identified by conventional lesion mapping.

## Introduction

Aphasia is a disorder of language processing that commonly results from acquired brain damage (Damasio, 1992) but the determinants of aphasia severity are not yet completely understood. Infarct volume is a well-defined predictor of severity, but it can only partly explain the degree of language problems (Pedersen et al., 1995; Forkel et al., 2014). Likewise, the cumulative amount of damage to language related cortical structures is another important determinant of severity, but it is still limited in its ability to define long-term impairments (Plowman et al., 2012). Some persons with aphasia with relatively intact language-related cortical structures may exhibit profound language deficits (Fridriksson et al., 2007), whereas others with more extensive damage display mild deficits (Epstein-Peterson et al., 2012).

This discrepancy may arise from white matter damage beyond the stroke lesion. Extensive damage to white matter has been well-documented after ischemic strokes (Matute et al., 2013): the white matter receives relative less cerebrovascular perfusion compared to gray matter (Fisher, 2011), and its anaerobic resistance declines with aging (Hamner et al., 2011). Furthermore, Wallerian degeneration from the cortical site of injury can lead to remote loss of structural connectivity (Kuhn et al., 1989; Thomalla et al., 2005; Wang et al., 2012; Jones et al., 2013). Interestingly, poststroke white matter disconnection of seemingly spared cortical structures can lead to dysfunction of the disconnected cortex that is tantamount to cortical necrosis (Bonilha et al., 2014). Several other studies have indicated an important role of white matter connections in shaping cognitive and language performance (Catani and ffytche, 2005; Catani and Mesulam, 2008; Kümmerer et al., 2013; Forkel et al., 2014; Duffau, 2015; Stockert and Saur, 2017). For this reason, to better understand the nature and magnitude of brain damage after strokes, it is important to take into account not only gray matter lesions, but also white matter pathways.

The structural human brain connectome is a novel methodological approach in clinical neurosciences. It entails mapping the entireness of medium to large-scale white matter connections in the brain, typically using diffusion tensor imaging (DTI) MRI, combined with high resolution anatomic images (volumetric T1- and T2-weighted scans). Our group has recently demonstrated that structural connectomes can be measured in brains with stroke lesions, and, more importantly, used to assess the relationship between regional network damage and language impairments in persons with aphasia (i.e., connectome lesion symptom mapping, CLSM). CLSM provides additional anatomic information regarding the neuronal networks whose lesions are statistically associated with impairments in behavior, evaluating damage more broadly defined as necrosis and disconnection.

Despite its advantages, CLSM based on connection weight does not provide information about network architecture beyond pair-wise connections. CLSM can identify which connections are crucial for behavior, but it is not sensitive to detecting the integrity of indirect connections. One possible method of addressing the problem of indirect pathways is the approach recently described by Sporns and colleagues (Mišić et al., 2015) to measure connectome dynamics. Their method leverages the structure of the connectome and evaluates the relative lag of transfer of information between different brain regions taking into account the presence and weight of direct and indirect connections. A faster spread on information is observed between regions that are linked by shorter path lengths, or by alternative nearly-shortest paths (the principal advantage of this approach over traditional path length).

This study had three aims. The first was to determine how much of the variance in aphasia severity can be explained by connectome dynamics lesion symptom mapping (CDLSM), CLSM or gray matter necrosis (regional based voxel-lesion symptom mapping). We tested the hypothesis that regional gray matter damage and network dysfunction are both crucial determinants of global aphasia and fluency impairments, using out of sample statistical predictions across a large cohort of chronic stroke survivors. The second aim was to test the hypothesis that combining connectome-based modalities (CLSM and CDLSM) with lesion damage improves prediction of aphasia severity. Finally, we aimed to disclose the cortical and subcortical networks related to aphasia severity and speech fluency.

## Materials and Methods

### Subjects

Ninety-two left hemisphere chronic stroke survivors (55 male, 37 female, mean age = 60.5 years, SD = 11.18 years), at least six months since the stroke, were included in this study. All participants were recruited through local advertisements and none had a premorbid history of other neurologic disorders affecting the brain or psychiatric disorders. All participants signed informed consent and the Institutional Review Boards at our institutions approved this study.

### Language testing

The presence and severity of aphasia was assessed using the Western Aphasia Battery-Revised (WAB-R; Kertesz, 2007), which was administered to all participants. Aphasia severity was determined using the WAB aphasia quotient (WAB-AQ). The WAB-AQ ranges from 0 to 100, with the cutoff for aphasia being set at as 93.8, and lower values indicate more severe aphasia. We also assessed speech fluency, as rated by clinicians using the WAB-R speech fluency rating scale, which ranges from 0 to 10 and lower values indicate less fluency.

### Image acquisition and postprocessing

Volumetric T1- and T2-weighted MRI sequences, as well as diffusion MRI, were obtained from all participants using Siemens 3T Trio Systems with a 12-channel head-coil. Scanning parameters were as follows: T1-weighted images: MP-RAGE sequence with 1 mm^3^ isotropic voxels, FOV matrix of 256 × 256 mm, 9-degree flip angle, and 192 sagittal slice sequence with TR = 2250 ms, TI = 925 ms, and TE = 4.15 ms, with parallel imaging (GRAPPA = 2, 80 reference lines); T2-weighted images: 3D SPACE, voxel size of 1 mm^3^, 256 × 256 mm FOV matrix, 160 sagittal slice sequence, variable flip angle, TR = 3200 ms, TE = 352 ms, with no slice acceleration. Slice center and angulation were similar to the T1 image sequence. Diffusion MRI: EPI scan using 30 directions with b = 1000 s/mm^2^ and b = 2000 s/mm^2^, TR = 6100 ms, TE = 101 ms, 82 × 82 matrix, 222 × 222 mm FOV, with parallel imaging GRAPPA = 2, 80 45 contiguous 2.7-mm axial slices, TA = 390 s. This diffusion sequences were acquired twice, and we also acquired an identical sequence with nine B = 0 volumes, yielding a total of 131 volumes (11 B = 0, 60 B = 1000; 60 B = 2000).

**Figure 1.**
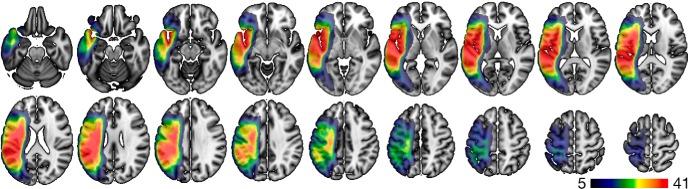
Voxel-wise lesion overlay, where each voxel is color coded in accordance with how many subjects had that voxel involved in the lesion. The color bar represents the number of subjects.

Lesion masks were drawn on the T2-weighted images by a neurologist who was unaware to the patient’s behavioral data at the time of the lesion drawing. A map of the lesion overlay is demonstrated in Figure 1. The T2 image and the lesion mask were coregistered to the T1-weighted image, which was normalized to standard space using an enantiomorphic approach to minimize the distortions caused by the brain lesion (Nachev et al., 2008) leveraging SPM12's (RRID:SCR_007037) unified segmentation-normalization, employing the lesion masks smoothed with a 3-mm full-width half maximum Gaussian kernel. The lesion masks were transformed into standard space and parcellated using an in-house anatomic atlas (described below) to determine the amount of damage to each cortical region, computed as the percentage of lesioned voxels. The inverse of the transformation matrix from T2/T1 space to standard space was used to transform the anatomic atlas into T2/T1 space, and subsequently into diffusion space, as described below.

### Anatomic atlas

To study the spread of information through the cortical network, it was important to use an atlas with high spatial resolution to define the nodes of the network with fine granularity. For this purpose, we created a cortical parcellation based on the AICHA atlas^14^. The AICHA atlas defines 384 gray-matter regions of interest (ROIs); we further subdivided these ROIs to obtain 1358 ROIs of approximately the same volume. The subdivision was conducted using the procedure described by Fornito et al. (2010), which allowed us to triple the number of ROIs while keeping the ROI size approximately constant, leveraging the fact that nodes with approximately the same volume reduces biases in streamline tractography.

### ROIs

**Figure 2.**
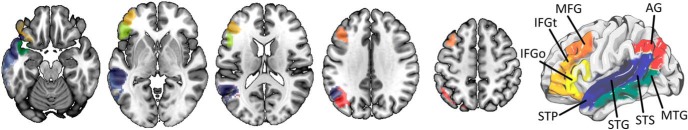
Language-specific ROIs used in this study. AG, angular gyrus; MFG, middle frontal gyrus; IFGt, IFG pars triangularis; IFGo, IFG pars opercularis; STP, superior temporal pole; STG, superior temporal gyrus; STS, superior temporal sulcus; MTG, middle temporal gyrus.

To reduce the probability of Type II statistical errors and focus on language relevant areas, all analyses were restricted to language-specific ROIs in the left hemisphere. These ROIs were derived from functional imaging studies by Fedorenko et al. (2012) and adapted for our parcellation. Specifically, eight ROIs were included: superior temporal gyrus, superior temporal sulcus, middle temporal gyrus, superior temporal pole, inferior frontal gyrus (IFG) pars triangularis, IFG pars orbitalis, middle frontal gyrus, angular gyrus (Fig. 2; Table 1).

**Table 1.** Language specific ROIs used in this study

ROI #	Name	AICHA regions
1	superior temporal gyrus	[652 655:657 661:668 676:682]
2	superior temporal sulcus	[689:691 693:696 704:709 716:721 729:734]
3	middle temporal gyrus	[739:741 744:748 754:756 759:762]
4	superior temporal pole	[815:817 820:821]
5	IFG triangularis	[160:168]
6	IFG orbitalis	[133:137 187 190:194 198:199]
7	middle frontal gyrus	[113:114 118:121 126:127]
8	angular gyrus	[400:404 410:413 419:421]

As such, for each individual, the degree of ROI damage corresponded to a 1 × 8 vector indicating the percentage of damage to each of the 8 ROIs. Likewise, connectivity analyses (explained below) were restricted to the subnetworks involving these eight ROIs.

### Structural brain connectivity

The whole-brain brain connectome was obtained using probabilistic DTI. Fiber tracking was performed in diffusion space. As described above, an enantiomorphic normalization was applied to transform the T1 into standard space, taking into account the deformations caused by the brain lesion. The inverse of this transformation matrix was then used to transform the anatomic atlas into T1/T2 space. The T2-weighted image was then linearly normalized into diffusion space (using the B0 image as reference) employing an affine registration with 12 parameters (Jenkinson et al., 2012). This registration matrix was used to normalize the probabilistic maps of white and gray matter (the latter divided into ROIs), and the lesion mask, into diffusion MRI space. Note that this last registration employed the same individual anatomy (from T2 to diffusion) and only a linear transformation was necessary. Once the anatomic atlas was transformed into diffusion space, pair-wise ROI connectivity was performed using FSL FDT’s probabilistic tractography method (RRID:SCR_002823; Behrens et al., 2007). FDT’s Bedpost built default voxel-wise distributions of diffusion parameters, based on the diffusion weighted data, the nondiffusion image and the gradient table. Bedpost was followed by probabilistic tractography (FDT’s probtrackX, with parameters: 5000 individual pathways drawn through the probability distributions on principle fiber direction, curvature threshold set at 0.2, 200 maximum steps, step length 0.5 mm, using distance correction built into probtrackx). The probabilistic white-matter map excluding the stroke lesion was used as a waypoint mask and the number of streamlines arriving in one ROI, when another ROI was seeded, was computed, averaging the connections from ROI_i_ to ROI_j_ and vice versa, and the number of streamlines was corrected based on distance between ROIs (from probtrackX, as mentioned above) and by the sum of volume of the ROIs (i.e., the resulting number from distance corrected probtrackX was divided by the sum of the volumes of the connected ROIs).

For each participant, a structural connectome including 1358 × 1358 connections was constructed (i.e., a 1358 × 1358 symmetrical adjacency matrix), where each connection represented the connection weight between ROIs (number of streamlines corrected by distance between ROIs and by the combined volume of connected ROIs). From this matrix, we extracted a subnetwork of 8 × 8 connections including only the language-specific ROIs described above (with 28 unique connections, since reciprocal connections were identical). This reduction in the number of connections was performed to focus on connections more commonly associated with language and reduce model overfitting.

**Figure 3.**
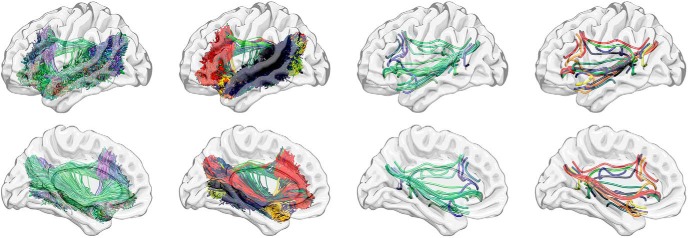
The connectivity weight analyses focused on a subnetwork of the whole brain connectome composed of all 28 unique possible connections between the eight language-specific ROIs. Each connection was independently assessed and their anatomic representations (in a cohort of healthy individuals; see Materials and Methods for details) are demonstrated in this figure. All deterministic streamlines are represented in the first column (colored in accordance with their main direction of displacement, as per tractography convention: red, lateral to lateral; blue, rostral to caudal; green, anterior to posterior). Each specific pair-wise connection is represented by a different color in the second column. The third column demonstrates the centers of mass (centroids) of each pair-wise connection (colored per tractography convention), and the fourth column demonstrates each connection centroid colored similarly to the second column. Note the comprehensive and intricate pattern of structural connectivity assessed in the connectivity weight analyses.

For the visual display of the anatomic subnetwork used in this study, we reconstructed the deterministic tractography streamlines between each pair of ROIs across 59 healthy controls with a similar age distribution [45 female, mean age 54.7 ± 8.3 years; DTI parameters: twice-refocused echo-planar imaging b = 0, 1000, 30 diffusion encoding directions, TR = 8500 ms, TE = 98 ms, FOV = 222 × 222 mm^2^, matrix = 74 × 74, 3-mm slice thickness, and 40 axial slices; DSI studio: fiber reconstruction using Q-Space Diffeomorphic Reconstruction (Yeh and Tseng, 2011), 1.25 diffusion length sampling ratio, 2-mm output resolution]. Each connection was transferred to stereotaxic space using 7, 9, and 7 transformation parameters (Fourier basis) and then combined across subjects, with the resulting bundle being interpolated into 100 segments and the center of mass for each segment was calculated. Streamlines whose segment-wise deviation from the center of mass was within 0.5 SD were maintained, yielding an anatomically representative connectome link. Likewise, the core pathway traversed by each connection was also maintained and used for visual display. to define the length of each pair-wise connection (i.e., the average length of the fibers constituting each connection), the distance between each 100 interpolated points was accrued for each link. The language network used in this study is illustrated in Figure 3.

### Structural brain dynamics

**Figure 4.**
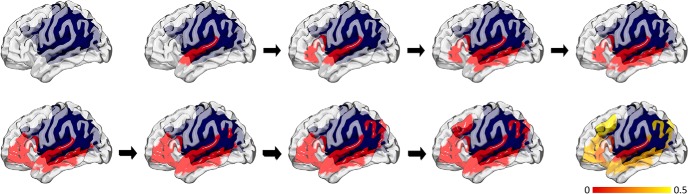
This figure illustrates the procedural steps used to assess connectome dynamics. The upper left subplot demonstrates a rendering of the cortical surface with the large cortical and subcortical poststroke lesion overlaid in blue. Next (second subplot on the first row), the superior temporal gyrus is seeded. Thereafter, based on the weight of structural connectivity between the superior temporal gyrus and the remaining whole brain connectome, the number of steps taken to reach each other language-specific ROI is calculated. The sequence of subplots demonstrated which ROIs are reached, in sequence. This process results in a vector denoting the inverse of the number of steps taken to reach each other ROI, when one ROI is seeded (illustrated in the bottom right, with the color bar illustrating the inverse of the number of steps). For each participant, this is repeated by seeding each ROI in turn, and 28 unique pair-wise connectome dynamics are calculated.

Connectome dynamics were extracted using the method described by Mišić et al. (2015). This method measures the spread of information throughout the network. It is performed by changing the state of one of the network nodes (“seeding” the network). In the next time step, the neighbors of the seed-node change their state, and in following time step, their neighbors change their state, and so on. While this approach is novel in the context of neuroimaging, it has a long and distinguished history in the social sciences (Granovetter, 1978; Watts, 2002). This process is illustrated in Figure 4. Unless the network contains unreachable nodes, all nodes eventually change their state. If the threshold is set to 0, the number of time steps that it takes to change the state of a node is equal to the length of the shortest path to the seed node.

Lesion disrupts the connectome and creates unreachable nodes; for numerical convenience, we used the inverse of the lengths of propagation paths (equivalent to the “speed” of propagation from the seed to the destination node). This way, the propagation speed between a pair of mutually unreachable nodes is zero (corresponding to the shortest path of infinite length). As a result, we obtained 28 values of propagation speed between each pairing of the eight language-network nodes.

Importantly, the pair-wise measures in the connectome dynamics matrix are a reflection of the structure of the whole network, thus providing information about the integrity of the topological organization of the network beyond the pair-wise connection. Conversely, the pair-wise connection weight is related exclusively to the connection between one pair of ROIs, respective of the other connections in the connectome.

### Statistical analyses and out-of-sample prediction

From the methods described above, five sets of variables were obtained from each participant: two behavioral measures, WAB-AQ and WAB fluency subscore; and three neuroimaging measures, ROI damage (1 × 8 vector of proportional damage to the eight language-specific ROIs), CLSM = connectivity weights (1 × 28 vector of the anatomic connection weights between each pairing of the eight language-specific ROIs), and CDLSM = connectivity dynamics (1 × 28 vector representing the connectivity dynamics within the language networks, i.e., the inverse of the shortest path length between each pairing of nodes).

We used linear-kernel support vector regression (SVR) to predict the behavioral measures (WAB-AQ and, separately, WAB fluency) from the neuroimaging measures (ROI damage, connectivity weight, connectivity dynamics) and an ensemble method that combined the three neuroimaging measures.

### Individual measures

The pipeline that predicted a behavioral measure from a neuroimaging measure consisted of SVR, followed by a third-order polynomial regression to match the range of the SVR predictions to the actual observed range. We used the LIBSVM implementation of SVR (Chang and Lin, 2011). The pipeline was trained and tested using 5-fold cross-validation. The four folds that composed the training set were also used to find the optimum regularization parameter for the SVR model (the term that defines the trade-off between margin width and training-set accuracy, known as the C value in the support-vector literature). This parameter was optimized using a grid search with values (.1, .5, 1, 10, 50, 100, 500). We have observed that SVR prediction tends to underestimate the highest scores and to overestimate the lowest scores; to correct for this, a third-order polynomial function was applied to the SVR-predicted scores. To compute the parameters of the polynomial, we predicted the scores of the patients in the training set (after the estimation of the SVR regularization parameter), and applied the least-squares regression to find the relationship between actual and predicted training scores (this is similar to the procedure described by Yourganov et al., 2016, except here we use a polynomial rather than a linear function). The pipeline predicted on the left-out fold and a correlation score was calculated between the predictions and truths. After all five folds served as the left-out fold, the five resultant correlations were averaged to form a single value. This was repeated multiple times to assess prediction distributions. K-fold cross-validation is known to have high variance (Ojala and Garriga, 2010), with results varying for the same dataset depending on the combinations of observations allocated among the folds. To correct for this instability, we performed 1000 iterations of 5-fold cross-validation (i.e., 1000 times the average from each 5-fold cross-validation described above), giving us 1000 values of correlation between actual and predicted scores. Of note, we did not perform classical mass univariate statistical analyses of lesions or connections. We employed these values to build the SVR model instead. For this reason, the SVR approach is not corrected by multiple comparisons in the classical sense of mass univariate statistics, but takes into account the multiple values in a multivariate approach and type 1 and 2 errors are reflected in reduced accuracy of the resulting model.

To statistically compare measures, we calculated the ratio of iterations that a particular measure had a higher mean correlation than another measure. This was possible because each measure was allocated the same folds at each of the 1000 iterations, although the allocation changed across iterations.

We also performed permutation tests to address the well-known issue that it is possible to obtain results that appear better than chance with small datasets that are randomly generated (Combrisson and Jerbi, 2015). For each measure, we calculated a permuted distribution by performing the same pipeline over 1000 iterations. However, the truth values used to train and test the model (i.e., the dependent variables WAB-AQ and WAB fluency) were shuffled at the beginning of each iteration. This resulted in a length 1000 permutation distribution for each measure. For each measure, each of the 1000 real correlations was compared with the permuted distribution, and had a *p* value calculated from its rank on the distribution. These 1000 *p* values were then averaged (Ojala and Garriga, 2010).

The coefficients of the linear SVR models were saved for each of the five folds and averaged. Each coefficient represents the relative predictive power of an individual feature. For the three types of input, the features were: damage to an ROI; strength of the anatomic connection between a pair of ROIs; the inverse of the shortest path between a pair of ROIs. By repeating the predictive process 100 times, we estimated the distribution of each coefficient.

### Combined measures

We created an ensemble pipeline that calculated a prediction for each subject by performing a weighted average on the subject’s corresponding predictions from ROI damage, connectivity weight, and connectivity dynamics.

The coefficients of the weighted average were found by fitting an ordinary least squares (OLS) multiple linear regression model. The OLS model consisted of three predictor (explanatory) variables corresponding to the behavior score predictions of the three neuroimaging-based models, and the prediction (response) variable was the behavior score. In other words, the model predicted a behavior score by combining behavior score predictions. OLS regression assigns larger weights for predictor variables that are more correlated with the prediction variable. Therefore, the neuroimaging-based models that more closely predicted the data had higher coefficients.

The four folds of the training set were used to fit the OLS model. To control for overfitting, we used a leave-one-out procedure (within the training set) to predict the scores using the three types of neuroimaging inputs and to combine them into a regression model. For computational reasons, the regularization parameters associated with the each of the neuroimaging models already found earlier for the training set were used to fit the models.

**Figure 5.**
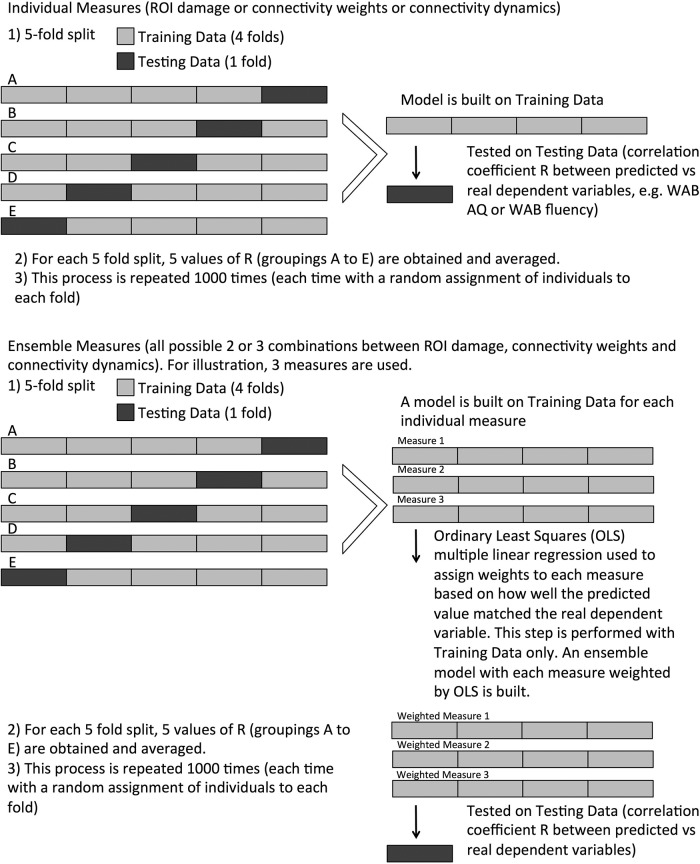
This diagram explains how the statistical analyses and out of sample SVR predictions were performed for models using individual measures or their combinations.

This pipeline was executed on 1000 iterations of 5-fold cross-validation, where the data allocation was the same as for the other measures. Just as the case of the individual measure-generated distributions, the resultant distribution was compared with the other measure’s distributions and with chance. A visual diagram of the statistical and machine learning steps used here is demonstrated in Figure 5.

### Software tools

The connectome dynamics were computed using the Matlab (RRID:SCR_001622) code developed by R. Betzel and B. Misic (Mišić et al., 2015). The predictive analysis was largely developed in the Julia and Python programming languages (RRID:SCR_008394; http://julialang.org). The Python library Scikit-learn (RRID:SCR_002577; Pedregosa et al., 2011) provided the SVR models, and the linear regression models used for the ensemble method and the third-order polynomial regression. The SVR models provided by Scikit-learn are based in LIBSVM (RRID:SCR_010243; Chang and Lin, 2011). The Julia code base communicated with Scikit-learn through the PyCall package. The visualizations were rendered with Matlab, Surfice, and MRIcroGL (RRID:SCR_008264, RRID:SCR_002403).

## Results

### Individual measures

Both WAB-AQ and fluency were predicted with an accuracy significantly higher than chance (*p* < 0.05). The mean correlation coefficients between the predicted and actual WAB-AQ values were: 0.760 ± 0.026 (combined methods), 0.723 ± 0.025 (ROI damage), 0.719 ± 0.025 (connectivity dynamics), and 0.534 ± 0.035 (connectivity weights).

**Figure 6.**
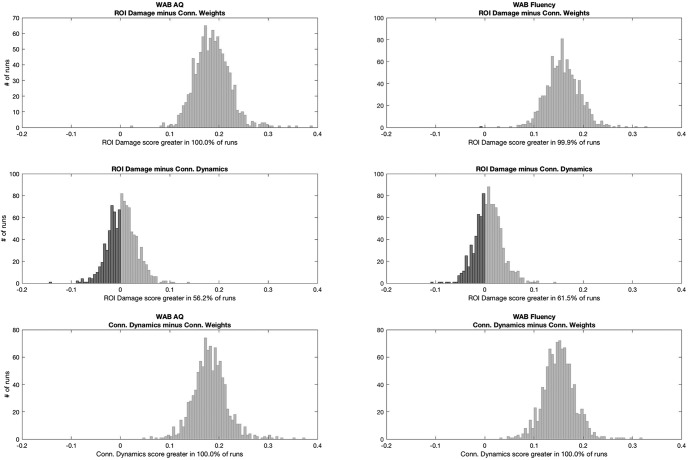
Statistical analyses comparing the distribution of Pearson correlation coefficients between real and predicted dependent measures for each model. Each subplot demonstrates the distribution of the subtraction of the correlation coefficient from one method minus another. This is possible since the training and testing split samples were identical for each model at every iteration, providing a direct comparison between models. If 95% of the subtractions felt above 0, the first test in the subtraction was considered statistically superior than the other at *p* < 0.05.

With regards to WAB fluency, the correlation coefficients were: 0.728 ± 0.023 (combined methods), 0.705 ± 0.026 (ROI damage), 0.697 ± 0.025 (connectivity dynamics), and 0.543 ± 0.030 (connectivity weights). ROI damage and connectivity dynamics were not significantly different from each other for predicting either WAB-AQ or WAB Fluency. However, both outperformed connectivity weights alone (Figure 6). Based on these results, connectome dynamics are as important as lesion location in predicting impairment. However, as explained below, Combined measures, the improved accuracy of the model based on their combination suggests that they provide additional and complementary information.

The ROIs with the highest influence on the predictive model based on ROI damage were, in descending order of importance: for WAB-AQ, superior temporal gyrus, superior temporal pole, angular gyrus, IFG triangularis, middle frontal gyrus, IFG orbitalis, middle temporal gyrus and superior temporal sulcus; for WAB fluency, superior temporal gyrus, angular gyrus, middle temporal gyrus, superior temporal pole, middle frontal gyrus, IFG triangularis, IFG orbitalis, superior temporal sulcus. These results are demonstrated in Figure 8.

**Figure 7.**
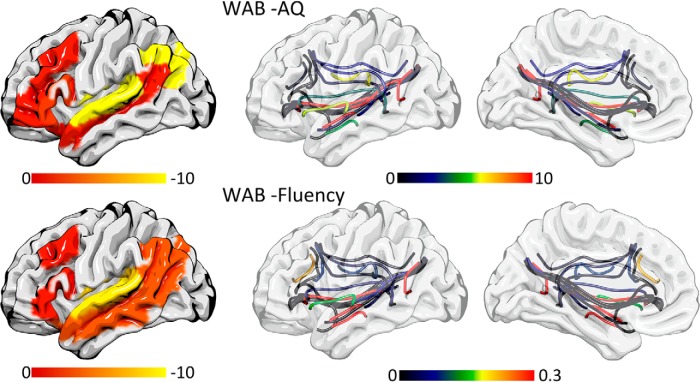
The individual coefficients are shown in color to illustrate which ROIs were more influential on the ROI model (left column) and which connections were more influential on the connectivity weights model (right most columns) for predicting WAB-AQ (first row) and WAB fluency (second row). The color bars indicate SVR coefficients.

The connections with the highest influence on the predictive model built with connectivity weights were: for WAB-AQ, superior temporal gyrus to superior temporal sulcus, superior temporal sulcus to middle temporal gyrus and superior temporal gyrus to superior temporal pole; for WAB fluency, middle temporal gyrus to superior temporal sulcus, superior temporal gyrus to superior temporal sulcus and superior temporal sulcus to angular gyrus (Fig. 7).

**Figure 8.**
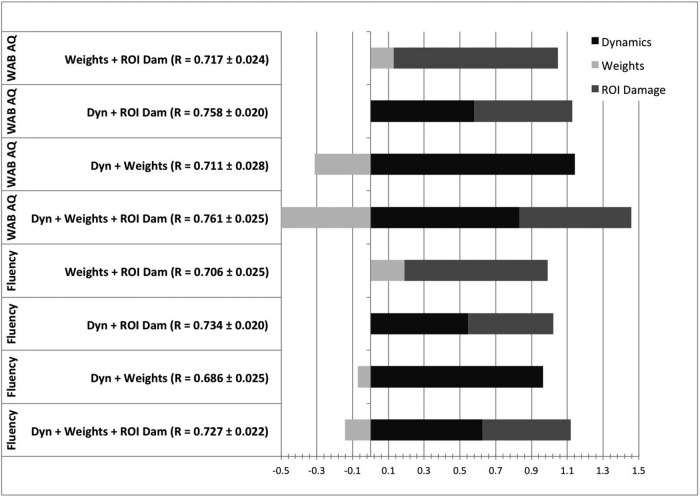
This graph illustrates the OLS weights assigned to each measure when models using combined measures were constructed (*x*-axis). For each possible combination, the correlation coefficients obtained with out of sample testing (i.e., when applied to the test data) are listed and the OLS weights are stacked to demonstrate their relative values.

On the model built based on connectivity dynamics, the connections with the highest influence toward WAB-AQ were superior temporal gyrus to angular gyrus, superior temporal sulcus to IFG triangularis, and superior temporal pole to angular gyrus. Toward WAB fluency: the superior temporal sulcus to IFG triangularis, middle temporal gyrus to superior temporal pole, and the superior temporal gyrus to IFG triangularis. The coefficients for the connectivity models are listed in Table 2.

**Table 2.** SVR Link-wise coefficients

Connection between		WAB AQ		Fluency	
ROI	ROI	Weight coefficients	Dynamics coefficients	Weight coefficients	Dynamics coefficients
IFG_orbitalis	angular_gyrus	0.12	2.99	0.01	-0.19
IFG_orbitalis	middle_frontal_gyrus	0.83	-1.01	0.46	0.26
IFG_triangularis	angular_gyrus	0.72	4.93	0.05	0.53
IFG_triangularis	IFG_orbitalis	5.93	2.06	0.86	0.19
IFG_triangularis	middle_frontal_gyrus	-0.55	0.92	0.32	0.59
middle_frontal_gyrus	angular_gyrus	1.47	6.25	0.12	0.92
middle_temporal_gyrus	angular_gyrus	5.89	8.76	0.97	0.27
middle_temporal_gyrus	IFG_orbitalis	0.40	2.05	0.04	0.66
middle_temporal_gyrus	IFG_triangularis	1.89	7.96	0.23	1.67
middle_temporal_gyrus	middle_frontal_gyrus	1.54	8.71	0.18	1.33
middle_temporal_gyrus	superior_temporal_pole	2.06	11.05	0.55	2.26
superior_temporal_gyrus	angular_gyrus	3.76	18.22	0.28	1.73
superior_temporal_gyrus	IFG_orbitalis	0.63	7.71	0.07	0.93
superior_temporal_gyrus	IFG_triangularis	6.02	12.44	0.81	2.07
superior_temporal_gyrus	middle_frontal_gyrus	1.02	6.20	0.11	0.83
superior_temporal_gyrus	middle_temporal_gyrus	1.48	10.72	0.22	0.48
superior_temporal_gyrus	superior_temporal_pole	9.13	-1.91	1.13	-1.14
superior_temporal_gyrus	superior_temporal_sulcus	13.11	8.32	1.59	0.09
superior_temporal_pole	angular_gyrus	1.15	12.59	0.08	1.83
superior_temporal_pole	IFG_orbitalis	-0.06	1.07	0.18	0.32
superior_temporal_pole	IFG_triangularis	1.89	4.48	0.68	0.52
superior_temporal_pole	middle_frontal_gyrus	0.23	6.14	0.02	1.15
superior_temporal_sulcus	angular_gyrus	5.17	-0.76	1.22	-0.25
superior_temporal_sulcus	IFG_orbitalis	0.31	1.18	0.05	-0.15
superior_temporal_sulcus	IFG_triangularis	2.35	13.80	0.40	2.53
superior_temporal_sulcus	middle_frontal_gyrus	2.45	6.13	0.30	0.91
superior_temporal_sulcus	middle_temporal_gyrus	11.34	8.69	1.84	0.66
superior_temporal_sulcus	superior_temporal_pole	1.45	3.43	0.89	-0.06

As expected, since WAB-AQ is a composite score that includes WAB fluency, there were strong correlations between WAB-AQ and WAB fluency (*R* = 0.92, *p* = 1.45E-39), and in the connection-wise coefficients related to WAB-AQ and WAB fluency (connectivity weights: *r* = 0.9, *p* = 3.54E-34; connectivity dynamics: *r* = 0.79, *p* = 5.06E-21), indicating that connections that were predictive of WAB-AQ were also likely to be predictive of WAB fluency. Nonetheless, there were no correlations between the connection-wise coefficients between connectivity weights and connectivity dynamics (WAB-AQ: *r* = 0.12, *p* = 0.61; WAB fluency: *r* = -0.3, *p* = 0.19), suggesting that links that were strongly influential in the predictive model built using connectivity weights were not necessarily influential in the model using connectivity dynamics.

It is important to emphasize that the model weights should be interpreted in the context of the SVR models (Haufe et al., 2014), and they have not been tested in a mass univariate approach to define the statistical significance of each feature. They are instead being reported in an ordinal order of importance with regards to how much each feature contributed to the model.

Interestingly, we observed an inverse linear relationship between connectome weight coefficients and the distance traveled by the fibers connecting ROIs (based on the normative data from control subjects; WAB-AQ: *R* = -0.46, *p* = 0.039; WAB fluency: *R* = -0.64, *p* = 0.001). However, there was not a relationship between dynamics coefficients and fiber distance (WAB-AQ: *R* = 0.23, *p* = 0.34; WAB fluency: *R* = 0.36, *p* = 0.11). Long connections had lower influence in the predictive model using connectivity weights, but not in the model built with connectivity dynamics. Of note, as expected, the distance traveled by the fibers connecting the ROIs was strongly but not perfectly correlated with the Euclidean distance between ROIs (*R* = 0.74, *p* = 7.27E-05) due to anatomic constraints on fiber trajectory, and the resulting fiber curvature.

### Combined measures

**Figure 9.**
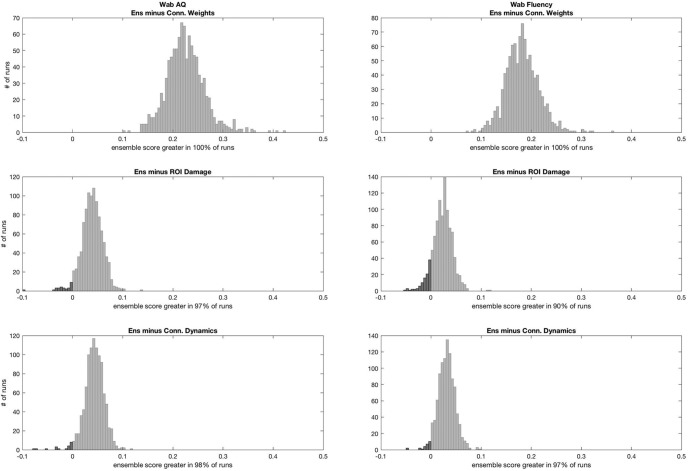
This figure illustrates the statistical comparisons between the model using all three measures (Ens, ensemble) versus each individual measure. Similar to Figure 5, the histograms demonstrate the distribution of the subtractions of the correlation coefficients from one method minus another for every possible run. If 95% of the subtractions fell above 0, the first test in the subtraction was considered statistically superior than the other at *p* < 0.05.

The highest predictive ability for WAB-AQ was observed with an ensemble model combining all three measures (ROI damage, connectivity weights, and connectivity dynamics; *R* = 0.761 ± 0.025) followed by the model combining connectivity dynamics and ROI damage. Regarding WAB fluency, the model with highest accuracy was composed of connectivity dynamics and ROI damage, followed by the model combining all three measures (*R* = 0.734 ± 0.02). The comparison between models is shown in Figure 9. Statistically, the model with three measures was significantly better at predicting language measures compared with the models based on ROI damage or connectivity dynamics alone: it outperformed ROI damage and connectivity dynamics when predicting WAB-AQ to a *p* value of 0.04 and 0.02, respectively. The ensemble performed better for predicting WAB fluency, but only to a *p* value of 0.08 (i.e., not statistically significant) but significantly better (*p* = 0.04) compared to ROI damage and connectivity dynamics, respectively (noting that *p* = 0.04 is weakly indicative of a rejection of the null hypothesis).

## Discussion

In this study, we evaluated the ability of neuroimaging methods to explain personalized severity of language problems across individuals with poststroke aphasia. We tested two specific hypotheses: (1) that connectome dynamics (CDLSM) could increase the correlation between residual connectome integrity and aphasia severity, i.e., provide a more comprehensive assessment of brain network integrity compared with CLSM; and (2) that combining connectome modalities with lesion damage would optimize the understanding of brain structures that are crucial for language and thus improve prediction. While testing these hypotheses, we also identified the crucial cortical and subcortical networks related to aphasia severity and speech fluency.

We observed that CLSM was statistically inferior to cortical ROI damage in predicting individual language deficits; however, CDLSM was comparable to cortical ROI damage and thus superior to CLSM. We also observed that the combination of measures was statistically better than each measure alone, supporting the hypothesis that each neuroimaging assessment may provide additional information about brain integrity and the crucial structures supporting language and fluency.

**Figure 10.**
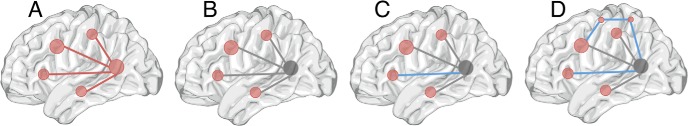
This diagram exemplifies the features evaluated by each modality. Considering a network of cortical structures (***A***), if one of the cortical regions is lesioned after a stroke, its connections are also affected (***B***; shaded gray node and gray lines). If all connections had a similar importance toward behavior (aphasia), CLSM would not distinguish between them. However, CLSM can identify which connections are more important (***C***; blue line). As such, CLSM is a subset of cortical lesion mapping. CDLSM, in turn, provides information about the direct and indirect connections that may be crucial for behavior (***D***; blue lines).

These results have broad implications for studies assessing crucial brain systems supporting cognitive function. First: how does CLSM complement classical lesion-symptom mapping using cortical damage? The results presented here suggest that CLSM has a lower personalized explanatory value toward aphasia severity compared with ROI damage. Therefore, is CLSM less well suited for behavioral mapping? It is important to consider that CLSM typically uses an exponentially larger number of predictive features compared with cortical ROI damage, since there are multiple possible combinations of ROIs. Thus, for the same number of individuals, the higher number of features in CLSM compared with ROI damage can lead to overfitting and reduce out of sample predictive performance. Likewise, CLSM is highly interrelated to cortical damage since damaged connections are likely those connecting lesioned ROIs. despite these caveats, we suggest that there are crucial features of CLSM that justify its use. CLSM can identify which connections are crucial within a set of all possible connections involving lesioned ROIs. As such, CLSM is a subset of conventional cortical lesion symptom mapping and refines the crucial network involving lesioned ROIs. Moreover, CLSM can also reveal the importance of connections involving lesioned ROIs and nonlesioned ROIs, thus elucidating the network extending beyond the cortical areas commonly injured due to vascular anatomy. If specific corticocortical connections did not influence behavior, all connections from a lesioned ROI would be equally predictors of outcome. Instead, there are crucial links that can be identified with CLSM, and the identification of which connections are crucial can improve the understanding of the systems underlying language production. A diagram representing this concept it presented in Figure 10.

Second, what does assessment of connectivity dynamics (CDLSM) add to ROI damage or connectivity weights? CDLSM is an expansion of CLSM; i.e., it requires the individual connectome, from which dynamic features can be extracted. CDLSM is unique because it can identify crucial corticocortical interactions that may occur indirectly even if their anatomic link is damaged. If the shortest path between nodes A and B is not through direct connections between A and B (because there is no structural connectivity remaining after the stroke), but rather through other nodes, CDLSM can measure this connectivity, while CLSM cannot. For this reason, it expands on CLSM, and provides a more comprehensive view of network integrity. CDLSM evaluates corticocortical interactions by taking into account shortest paths, even if the shortest path involves remote unaffected areas, such as the contralateral hemisphere or subcortical gray matter such as basal nuclei. Therefore, each link in CDLSM is a reflection of the topological organization of the entire network. This characteristic of CDLSM is particularly relevant in cases on neurologic recovery, where not only what was damaged during the lesion, but what was left after it, is crucial to the severity of chronic deficits. As demonstrated here, CDLSM is equivalent to cortical ROI damage with regards to out of sample personalized aphasia severity prediction. Importantly, our results also suggest that the combination of modalities yields the best personalized explanatory features, confirming that CLSM and CDLSM add additional information to mapping based on cortical damage and thus provide additional information about brain integrity that this crucial for aphasia. It should be mentioned that the connectivity dynamics approach used in this study, described by Mišić et al. (2015), is not the only approach to assess dynamics or communication within networks. Other alternatives include communicability (Crofts et al., 2011), search information (Goñi et al., 2014) and K-shortest paths (Avena-Koenigsberger et al., 2017), and these were not tested in this present study. Furthermore, it is important to emphasize that CLSM utilizes more basic edge-level information, and it does not take into account other topological features of network organization that can be obtained, for example, with graph theory. Likewise, CLSM used here employs number of streamlines, which is a measure that can vary due to signal-to-noise and method of tractography. A recent review article by Aerts et al. (2016) has provided a comprehensive overview of several studies that have investigated topological network changes associated with brain injury and strokes, with some few studies using DTI data. Our group also demonstrated in previous studies that temporal lobe betweenness centrality (Bonilha et al., 2016), cortical hub (“rich-club”), status (Gleichgerrcht et al., 2015), and global network small-worldness (Bonilha et al., 2016) are associated with aphasia severity and recovery. Given the plethora of possible measures, there are several possible combinations that have not yet been systematically assessed, and compose an interesting pathway for future studies. Furthermore, these methods could also be expanded to combined with connectomes obtained from functional MRI data (task based or resting state), with the caveat related to potential changes in hemodynamic properties and their effect on BOLD signal in stroke survivors with multiple cardiovascular risk factors and atherosclerosis (D'Esposito et al., 2003). Special attention should be paid to inferences about functional imaging (and neuroimaging in general) in the context of pathologically altered cerebral perfusion patterns (Siegel et al., 2017). In a recent and elegant study, Pustina et al. (2017) demonstrated that combining functional connectivity with structural connectivity increases the ability to explain and predict specific language deficits in chronic poststroke aphasia. They concluded that predictions based on multimodal data were more accurate than predictions using only one modality. It is reassuring that our results, i.e., improved prediction when combining CLSM and CDLSM with lesion based mapping, are similar to their conclusions, even if we used a different approach based only on structural integrity. Overall, their and our findings suggest that there is additional information from each modality that can leveraged to better understand the neurobiology of poststroke impairments and aphasia. Importantly, our findings suggest that CDLSM is particularly useful in improving predictions, indicating that connectome based studies, which are instrumental in expanding the understanding of the relationship between brain structural integrity and behavior (Yourganov et al., 2016; Gleichgerrcht et al., 2017; Marebwa et al., 2017), can be even further improved by including connectome dynamics. To our knowledge, this is one of the first studies to assess structural connectome dynamics related to stroke recovery.

Third, how did the results shown here, using additional modalities (CLSM and CDLSM) expand on the knowledge about aphasia severity and fluency impairment in aphasia? As expected, they revealed corticocortical interactions within the typical lesion bed are strongly predictive of chronic deficits. Notably, they demonstrated the crucial importance of ventral stream (Fig. 7). Moreover, they also demonstrated that connectivity structure and dynamics involving the angular gyrus had a strong influence in the model predicting WAB-AQ and QAB fluency, suggesting that the integrity of posterior temporal-parietal network plays a crucial role in aphasia severity. The posterior temporal-parietal is a watershed zone for the middle cerebral artery and the degree of cortical damage in this region may be unrelated to the degree of white matter damage, particularly given the decreased resilience of white matter to ischemia (Fisher, 2011; Hamner et al., 2011). As such, by elucidating this crucial component of the language networks, CDLSM can complement ROI damage and thus improve personalized prediction of severity. We propose that this approach should now be used to also evaluate the personalized mechanisms supporting spontaneous or treatment-related aphasia recovery.

One limitation of this study is that the measures of aphasia and fluency severity are imperfect. Not only is the WAB-AQ a multidimensional measure, but the fluency score also reflects multiple, distinct aspects of speech “fluency,” including grammaticality, articulation, hesitancy, among others. Furthermore, the inter-rater reliability in scoring is not high, because it is both subjective and multidimensional (Trupe, 1984). However, a more reliable and unidimensional score would likely have a higher, rather than lower, correlation with specific lesions and connections. Moreover, the specific measures of severity are not especially important for achieving our goal of evaluating the contribution of CDSLM to cortical necrosis and CLSM in predicting severity of language impairment.

## Synthesis

Reviewing Editor: Morgan Barense, University of Toronto Decisions are customarily a result of the Reviewing Editor and the peer reviewers coming together and discussing their recommendations until a consensus is reached. When revisions are invited, a fact-based synthesis statement explaining their decision and outlining what is needed to prepare a revision will be listed below. The following reviewer(s) agreed to reveal their identity: Daniele Marinazzo.

The reviewers and I are in agreement that your paper addresses a timely issue and that your novel approach has the potential to significantly advance the field. However, both reviewers have raised some concerns, which will be important to address in your revision. Below you will find the reviewers' specific comments. I hope you find these helpful as you prepare your revision.

REVIEWER 1

Dear authors

Thanks a lot for submitting your work. I find your study very timely, since it addresses an approach that is surprisingly lagging behind in clinical applications.

A small bunch of other studies have been conducted on a network approach to stroke lesions, measuring different quantities and reporting different results, as summarized in a recent review (Aerts et al., Brain 2016 https://academic.oup.com/brain/article/139/12/3063/2629972/Brain-networks-under-attack-robustness-properties). It would be interesting if you could give your opinion on the most convenient and robust network measures in this context.

Since stroke affects vascularization, is it possible that the blood perfusion, and consequently the BOLD signal, is changed across the whole brain, with an extent depending on the size and location of the stroke?

Also, could you please comment on the choice of standard versus subject space in this context?

P values are reported without an effect size, and are dychotomized (smaller or greater than 0.05). On the other hand p values are a continuous set, and all values should be reported, together with their effect size, to facilitate re-analysis and meta-analysis.

Also, it is not clear whether and how these values were corrected for multiple comparisons.

If you cannot share the raw data, please consider uploading the unthresholded statistical maps and atlases to neurovault.org to facilitate future re-analyses and meta-analyses.

REVIEWER 2

This paper explores the relationship between aphasia severity and the pattern of lesions and white-matter disconnections. Previous studies have explored the link between lesions and aphasia types, or disconnections but few have attempted to combine the information. This study does so using SVR models.

Introduction

I agree that there has been less focus on white matter connectivity in the aphasia literature, but I think it is unfair to say we don't have any prior knowledge. There are a number of studies, both historic (Geschwind, Wernicke), recent imaging (i.e. Saur and Catani groups) and invasive stimulation (i.e. Duffau), that have pointed to the involvement of multiple white matter connections. These are not discussed.

I am not sure about the phrase (pg4 line 14-15) “CLSM can identify which connections are crucial for behaviour”. I think the previous analysis have shown that certain patterns of connection were related to behaviour, but I not sure if it suggests which connections in the range of connections are ‘crucial’ for behaviour. This phrase indicates some causative link, but these analyses identify a collection of connections which are correlated with behaviour and do not test the importance of each singly. One would only be able to test this empirically by disrupting each connect separately.

Some other important differences with Yourganov et al include - the use of 8 language-related ROIs means that the model is forced to consider longer-range connectivity (Yourganov et al typically selected short connections around the edge of the lesion as being functionally correlated)

Methods

It is not clear why the DWI data were collected twice and why there were 9 B0 images. Typically, one would collect the data in opposing phase encoding directions but there is no mention of this. It is not clear what pre-processing was done to the DWI data (i.e. top-up field correction or eddy-current correction), it only states that it was put through FSLs BEDPOST algorithm.

A very fine atlas was generated but then only a sample of ROI were used - thus I am not sure why the detailed atlas is mentioned as only the coarser ROI set was utilised. I presume the full atlas was down-scaled due to computational issues and/or overfitting.

Rather than down-scaling the full atlas in an even way, only a set of 8 ‘language’ perisylvian ROIs were used. This means that any regions (and their connections) were not considered in the model.

Why were the images only normalised using a linear transformation? Did the more typical additional nonlinear components not work (cf. normalising a brain with a lesion)?

(p.8) - Number of streamlines is often avoided by researchers who use probabilistic tractography because the value is not a direct reflection of connection strength between seed and target regions; it is influenced by many other factors including crossing and forking fibres, distance, size of ROIs, etc.

“number of streamlines was corrected based on distance between ROIs and by the sum of volume of the ROIs.” - exactly how was this done? A number of research groups (e.g., Derek Jones, Cardiff) have proposed various possibilities.

Computing a full 1358^2 matrix is very impressive (the computational processing time must have been very time-consuming) but, unless I have misunderstood, the authors actually use an 8^2 (symmetrical) matrix. Having gone to the trouble and undoubted headache of computing the very large matrix, readers will wonder why such a small matrix was used in the analyses? Is it because there were too many predictors on the SVR (even with regularisation)?

p.10, line 8: The paragraph starting ‘Using the weighted connectome graphs ...’ has agrammatic and incomplete sentences and is almost impossible to understand.

WAB severity and fluency were the selected behavioural measurements. Were they correlated with each other? Note this might explain why the results were so similar.

SVR interpretation - (pg13 line 4): note the weight values in SVR (and related models) are not necessarily related to predictive power (see Haufe et al., 2014 on interpreting weights). In general terms, all the features are contributing in some way to the prediction.

Results

It would be helpful to display a lesion overlap map for the cases included in this study. This now seems to be standard practice for studies of this type.

p.15, line 6: I think this result is an important one - it seems that the model with lesion alone is better than any of the connectivity models and is as good as any of the combined models. This is a potentially important finding and needs discussion.

p.15-6: my understanding of SVRs is that the size of the weights is not necessarily related to their importance (Haufe et al 2014). This is because all of the predictors in the model are non-independent. My understanding is that currently, there is no mathematical way to assess the individual predictors in any of these machine-learning models.

Overall, I wonder if damage away from the core MCA territory might be responsible for the pattern of weights? For example, more severe cases have larger lesions away from core MCA territory and therefore impact regions such as AG, pMTG, STS

Discussion

The opening of the Discussion and Abstract suggests that the combined model is better than lesion alone. However, whilst numerically true, it does not appear to be significantly better.

One issue with the comparison of models is with providing an outcome measure that is corrected for multiple inputs (i.e. adjusted goodness of fit value). It is hard to determine if the combined model (with 3 predictors) is actually better than another model (with 1 or 2 predictors), if the more complex model is not penalised for complexity.

The dynamics measure is complicated. If it is a distance metric, then surely shorter connections are more likely to be affected and/or co-occur together vs. distant connections? Based on Fig. 9D I am not sure how the data speak to this description of finding direct and indirect connections. The metric tells you about the shortest length between nodes, so if there are indirect connections that are not in the node space, then these will be missed in the current analysis.

Minor:

The hyphens in line 10 of the abstract were difficult to parse.

## Author Response

Point by point response letter

REVIEWER 1

Dear authors

Thanks a lot for submitting your work. I find your study very timely, since it addresses an approach that is surprisingly lagging behind in clinical applications.

A small bunch of other studies have been conducted on a network approach to stroke lesions, measuring different quantities and reporting different results, as summarized in a recent review (Aerts et al., Brain 2016 https://academic.oup.com/brain/article/139/12/3063/2629972/Brain-networks-under-attack-robustness-properties). It would be interesting if you could give your opinion on the most convenient and robust network measures in this context.

Response: We appreciate the reviewer's enthusiasm towards our study and we welcome the suggestion. Aerts et al. is indeed a superb article, in which several networks measures are cited and discussed.

Since connectome based assessments constitute a novel approach to evaluate neurological improvement, the full extent to which each network measure can inform about stroke recovery still remains to be defined. Furthermore, there is a methodological difference concerning whether the network measure was obtained from structural measures (i.e., MRI DTI tractography) or from functional imaging (fMRI BOLD). As the reviewer knows, connectome networks can be reconstructed from both measures, and albeit being inter-related, they do not illustrate the same biophysical process.

Thus, we can offer our opinion on the nascent data regarding language and recovery and how they relate to network measures, including work from our group. For example, we recently demonstrated that temporal lobe network integration was associated with treatment-related recovery from chronic aphasia (Bonilha et al., 2016). Specifically, betweenness centrality was the measure of integration more closely related to improvement in naming. Interestingly, in the same study, we observed that recovery was also associated with a global network measure of small worldness (which is the balance between integration, i.e., global path length, and segregation, i.e., global clustering coefficient). Similarly, on another study, we observed that loss of hub status of key cortical regions is associated with aphasia severity (Gleichgerrcht et al., 2015). In addition to graph theory measures, we have also observed that pure connectome edge weight can be a significant determinant of behavioral performance (Yourganov et al., 2016) .

These are only the results from our group. As comprehensively and expertly summarized in the above article lead by Danielle Marinazzo, several studies have investigated topological network changes associated with brain injury and strokes (Aerts et al., 2016), with some few studies using DTI data. Notably, Crofts et al. demonstrated decreased communicability in structural networks following a stroke (Crofts et al., 2011). However, Schmitt et al recently noted that structural network parameters were not good predictors of functional imaging activation in experimental models(Schmitt et al., 2017).

Overall, it is important to consider that four main types of overlapping information can be obtained from connectome networks: node data, edge data, subnetwork data, and global network data. From each level, in addition to basic strength, several graph theory properties can be derived to explain the balance between integration and segregation into the remaining network. Furthermore, as described in our article, connectome dynamics can also be simulated from structural or functional data, adding a new dimension to the aforementioned data. Thus, given the plethora of possible measures, there are several possible combinations that have not yet been systematically assessed, and compose an interesting pathway for future studies. In our article, we have used information at basic levels, i.e., edge weight and connectome dynamics. We have now expanded the discussion to emphasize some of the points discussed above and also to highlight the potential for combining information in the future to optimize the potential of connectome studies to disclose the mechanisms underlying stroke recovery, and potentially serve as a clinical tool. (Pages 21 and 22)

Since stroke affects vascularization, is it possible that the blood perfusion, and consequently the BOLD signal, is changed across the whole brain, with an extent depending on the size and location of the stroke?

Response: This is a very important point. Many stroke survivors have many cardiovascular risk factors, which can lead to large and small vessel atherosclerosis, microangiopathic changes, and lypohialinosis. All of these can lead to significant changes in brain vasculature and brain perfusion. They may greatly affect MRI contrast and BOLD signal, notably with changes in the hemodynamic response function. These are important limitations of using functional MRI to reconstruct the connectome. As the reviewer noted, our study was performed exclusively with structural connectomes obtained from DTI MRI. We have now explained that our approach could be used to functional connectomes, but caution should be used to interpret that results given the limitations of functional imaging in the case of cardiovascular risk factors. (Page 22)

Also, could you please comment on the choice of standard versus subject space in this context?

Response: It is important to emphasize that the connectome, i.e., the connections between brain regions, is calculated in the subject's diffusion space. This is done to avoid distorting the diffusion image and thus disrupting fiber tracking, which can occur when the diffusion images are transferred to other spaces. However, it is also important to emphasize that transformations to and from the standard space are crucial to enable the transformation of brain regions from atlases (in standard space) to diffusion space, where tractography is performed. More specifically, the brain regions are first transferred to T1 weighted subject space, and trimmed to fit the subject's gray matter (based on the probabilistic gray matter map), and then moved into the diffusion space. Once the connectome is calculated, group level analyses can be performed in ‘connectome space’ since each matrix entry corresponds to the same anatomical connection. We have clarified these points in our study as suggested. (Pages 6, 7, 8 and 9)

P values are reported without an effect size, and are dychotomized (smaller or greater than 0.05). On the other hand p values are a continuous set, and all values should be reported, together with their effect size, to facilitate re-analysis and meta-analysis. Also, it is not clear whether and how these values were corrected for multiple comparisons.

Response: We appreciate the reviewer's suggestion. We have now avoided dichotomizing the p values and we report their specific values (Pages 16, 17 and 18). In this study, we did not use classic mass multivariate symptom mapping, where one statistical test is applied for each voxel or each edge multiple times, with a high probability of type 1 statistical errors, and thus a strong need for corrections for multiple comparisons. Instead, for each modality (lesion, CLSM, CDLSM), we built a multivariate model including and tested its accuracy with machine learning. Thus, this is the reason why corrections for multiple comparisons were not used, since type I and type II errors would be reflected in poorer accuracy of the model. We have also clarified this topic in the text accordingly. (Pages 12 and 13)

If you cannot share the raw data, please consider uploading the unthresholded statistical maps and atlases to neurovault.org to facilitate future re-analyses and meta-analyses.

Response: We agree with sharing our raw connectome data in neurovault.com once the article is ‘in press’ for publication. We agree with the importance of sharing data to improve scientific rigor and transparency, to promote reliability studies and foster future meta-analyses. If our paper is accepted for publication, we will add a statement describing the data being shared (i.e., the lesion vectors, the connectome maps [CLSM and CDLSM] and the WAB scores).

REVIEWER 2

This paper explores the relationship between aphasia severity and the pattern of lesions and white-matter disconnections. Previous studies have explored the link between lesions and aphasia types, or disconnections but few have attempted to combine the information. This study does so using SVR models.

Introduction

I agree that there has been less focus on white matter connectivity in the aphasia literature, but I think it is unfair to say we don't have any prior knowledge. There are a number of studies, both historic (Geschwind, Wernicke), recent imaging (i.e. Saur and Catani groups) and invasive stimulation (i.e. Duffau), that have pointed to the involvement of multiple white matter connections. These are not discussed.

Response: We agree with the reviewer that the existing literature was suboptimally discussed. While connectome based studies of stroke survivors are not very numerous, there are indeed several other important contributions to the literature regarding structural connectivity that we did not discuss in more detail. It was not our intention to exclude these studies and we apologize for not including them. We have now expanded the introduction to better address this prior work. (Pages 3 and 4)

I am not sure about the phrase (pg4 line 14-15) “CLSM can identify which connections are crucial for behaviour”. I think the previous analysis have shown that certain patterns of connection were related to behaviour, but I not sure if it suggests which connections in the range of connections are ‘crucial’ for behaviour. This phrase indicates some causative link, but these analyses identify a collection of connections which are correlated with behaviour and do not test the importance of each singly. One would only be able to test this empirically by disrupting each connect separately. Some other important differences with Yourganov et al include - the use of 8 language-related ROIs means that the model is forced to consider longer-range connectivity (Yourganov et al typically selected short connections around the edge of the lesion as being functionally correlated)

Response: We agree with the reviewer's comment. Our original intention was to provide a parallel with lesion symptom mapping whereby cortical regions are typically considered crucial for behavior when the behavior is disrupted if that region is lesioned. In both cases, i.e., lesion symptom mapping and CLSM, the relationship between function and structure is strong, but cannot determine for certain if the lesioned area (or connections) is involved with the behavioral function; it could be linked to the behavioral through a broader constellation of regions or connections. We also agree with the difference highlighted between this study and Yourganov et al. We have removed this sentence and clarified these points in the text accordingly. (Page 4)

Methods

It is not clear why the DWI data were collected twice and why there were 9 B0 images. Typically, one would collect the data in opposing phase encoding directions but there is no mention of this. It is not clear what pre-processing was done to the DWI data (i.e. top-up field correction or eddy-current correction), it only states that it was put through FSLs BEDPOST algorithm.

Response: We thank the reviewer for the insightful comments. These scans were acquired on clinical systems where the possibility to insert own gradients tables was limited, and before modern methods using reversed phase encoding gradients were developed. Therefore, to increase the number of B=0 scans we needed to acquire images in multiple sessions. We do wish to note that these sequences were tuned to reduce many of these spatial distortions, for example the modest matrix size (for rapid EPI readout), and use of a twice-refocused spin echo sequence (which reduces distortion at the expense of SNR). These sequences reflect a careful compromise with scanning time. While these limitations may reduce our statistical power, we wish to point out that our manuscript focuses on positive findings despite these limitations. No additional steps beyond those described in the manuscript were performed to process tractography. We clarified that FSL Bedpost requires the 4D data with a separate no diffusion 3D image, a brain mask and gradient table. (Page 8)

A very fine atlas was generated but then only a sample of ROI were used - thus I am not sure why the detailed atlas is mentioned as only the coarser ROI set was utilised. I presume the full atlas was down-scaled due to computational issues and/or overfitting. Rather than down-scaling the full atlas in an even way, only a set of 8 ‘language’ perisylvian ROIs were used. This means that any regions (and their connections) were not considered in the model.

Response: This is a cumulative dataset and the very fine atlas was generated to enable the reconstruction of networks or subnetworks with greater or lesser degree of anatomical precision based on goals of different studies. Since our goal was to compare modalities (CLSM, CDLSM, etc), we opted to assess robust and down scaled networks focusing on language specific regions to maximize their relationship with language and to avoid overfitting (and type II error), as the reviewer suggested. We have now clarified this point in the text accordingly. (Page 9)

Why were the images only normalised using a linear transformation? Did the more typical additional nonlinear components not work (cf. normalising a brain with a lesion)?

Response: We apologize if this topic was not clear in the text and we have now re-written it to better explain how normalization was done. Specifically, a non-linear normalization was applied to transform the regions of interest from the standard space to the individual space (T1 and T2 images). This was performed using an enantiomorphic approach for focally lesioned brains (Nachev et al., 2008), which is a special approach to avoid distortions caused by the stroke lesion, and can be understood as an improvement over classical cost-function masking (Brett et al., 2001). The enantiomorphic approach was used to convert the T1 images into the standard space, and the inverse of the resulting transformation matrix was used to transform the atlas into T1 space. The T2 image was resliced into the T1 image, so the T1 space was the same as the T2 space. Once the regions of interest were in the individual's own space (T2 image), then they were non-linearly registered into the diffusion space. This last transformation was performed in a non-linear fashion since the brain lesion can be well matched between T2 and B0 images, since it is the same subject. Subsequently, all tractography calculations were performed in diffusion space. We apologize for the lack of clarity in the description of the non-linear parameters and the special attention to the brain lesion and we have now clarified these points accordingly. (Pages 6, 7 and 8)

(p.8) - Number of streamlines is often avoided by researchers who use probabilistic tractography because the value is not a direct reflection of connection strength between seed and target regions; it is influenced by many other factors including crossing and forking fibres, distance, size of ROIs, etc. “number of streamlines was corrected based on distance between ROIs and by the sum of volume of the ROIs.” - exactly how was this done? A number of research groups (e.g., Derek Jones, Cardiff) have proposed various possibilities.

Response: We agree with the reviewer that using number of streamlines is an approach with limitations, both for probabilistic tractography, as well as for deterministic tractography. This is possibly one of the reasons why CDLSM had an additional value is contributing in the prediction model. We also agree that there are other reasonable approaches besides number of streamlines, including progressively higher fixed density binary networks, as we, and others, have used in the past (Bonilha et al., 2012). However, these methods are not well suited to quantify edge weight. Likewise, edge weight measured through deterministic tractography is likely limited by deterministic tractography suboptimal ability to resolve crossing fibers or areas of high curvature. Thus, there is a trade-off between limitations and we have opted to used edge weight from probabilistic tractography, given that we were also comparing this method with lesion and CDLSM, and therefore benchmarking it against other approaches. We have discussed this point in the text accordingly now. (Page 21) We have also clarified that distance correction was used based on the function inbuilt in FSL Probtrackx, and the correction based on the size of the regions of interest was performed by dividing the number of fibers by the sum of the volumes of the connected regions. (Page 9)

Computing a full 1358^2 matrix is very impressive (the computational processing time must have been very time-consuming) but, unless I have misunderstood, the authors actually use an 8^2 (symmetrical) matrix. Having gone to the trouble and undoubted headache of computing the very large matrix, readers will wonder why such a small matrix was used in the analyses? Is it because there were too many predictors on the SVR (even with regularisation)?

Response: The reviewer is correct. We opted to focus the analyses on a subnetwork with a more direct involvement with language, to maximize the relationship between brain structure and function, and to avoid overfitting. As explained in the answer to the question above, this is a continuous dataset which was acquired and processed to enable multiple parcellations. We did not explore various other forms of parcellation in our study as we felt that this would be beyond the scope of this study.

p.10, line 8: The paragraph starting “Using the weighted connectome graphs ...” has agrammatic and incomplete sentences and is almost impossible to understand.

Response: We apologize for the lack of clarity and we have now re-structured this paragraph. (Page 11)

WAB severity and fluency were the selected behavioural measurements. Were they correlated with each other? Note this might explain why the results were so similar.

Response: Yes. That is an important point. Fluency and severity are often closely associated, even though there are subtle differences between the measures. We have now reported their correlation (R square = 0.84, p>0.0001) and explained this point accordingly. (Page 16)

SVR interpretation - (pg13 line 4): note the weight values in SVR (and related models) are not necessarily related to predictive power (see Haufe et al., 2014 on interpreting weights). In general terms, all the features are contributing in some way to the prediction.

Response: The reviewer is correct. The weights are only interpretable in the context of the model. One possible approach to assess the contributory value of each weight is to keep all other weights constant and assess whether changing the weight of each predictor significantly affects the overall resulting model. This is a very computationally intensive and time consuming approach. Since our goal was to assess models (CLSM vs CDLSM), we believe that this approach was beyond the scope of this article. We have now clarified these points accordingly. (Page 17)

Results

It would be helpful to display a lesion overlap map for the cases included in this study. This now seems to be standard practice for studies of this type.

Response: We have now provided a new supplementary figure with the lesion overlap as requested. (Pages 6 and 7, Figure 1).

p.15, line 6: I think this result is an important one - it seems that the model with lesion alone is better than any of the connectivity models and is as good as any of the combined models. This is a potentially important finding and needs discussion.

Response: We agree. We also want to emphasize that ROI damage and connectivity dynamics were not statistically significantly different from each other for predicting either WAB AQ or WAB Fluency. However, both outperformed connectivity weights alone. The main implication of this finding is that connectome dynamics is as important was lesion location in predicting impairment. Moreover, and more importantly, they likely represent different information since the combination of their data outperforms. The highest predictive ability for WAB-AQ was observed with an ensemble model combining all three measures. We have now expanded this discussion and clarified this point accordingly. (Page 16)

p.15-6: my understanding of SVRs is that the size of the weights is not necessarily related to their importance (Haufe et al 2014). This is because all of the predictors in the model are non-independent. My understanding is that currently, there is no mathematical way to assess the individual predictors in any of these machine-learning models.

Response: We agree. Please note the response the similar question above. We have now clarified that the weights are interpretable only in the context of the models.

Overall, I wonder if damage away from the core MCA territory might be responsible for the pattern of weights? For example, more severe cases have larger lesions away from core MCA territory and therefore impact regions such as AG, pMTG, STS

Response: The reviewer brings an important point, which we believe can be clarified by the new image showing the lesion overlap. The voxel-wise demonstrates that the AG, pMTG, STS are very close to the core of the lesion locations. Larger lesions tend to affect more peripherally located regions such as the temporal pole, superior frontal gyrus, etc. (Figure 1)

Discussion

The opening of the Discussion and Abstract suggests that the combined model is better than lesion alone. However, whilst numerically true, it does not appear to be significantly better.

Response: We agree that this point was not clear in our original paper. The combined model is statistically significantly better than the other models and this is demonstrated in Figure 9. (The reference to this figure was missing in the text and this probably caused the lack of clarity). In brief, we repeated the model several times and assessed the number of times one approach (for example, combined model) outperformed the other approach (i.e., had a numerically higher correlation coefficient between predicted and actual scores in the testing data). If the probability of outperforming was higher than 95% of the times (i.e., 5% or less of error in rejecting the null hypothesis), then one approach was considered statistically superior. This is a relatively conservative approach to test superiority between machine learning models (as opposed to just comparing the numerical accuracy). We have clarified this topic in the text accordingly. (Page 18)

One issue with the comparison of models is with providing an outcome measure that is corrected for multiple inputs (i.e. adjusted goodness of fit value). It is hard to determine if the combined model (with 3 predictors) is actually better than another model (with 1 or 2 predictors), if the more complex model is not penalised for complexity.

Response: The reviewer brings an important point. It is very likely that a model with many predictors is penalized by the higher number of variables and the possibility of over fitting, and by the inter relationship between its variables. In this case, the combined model would be penalized. Our attempt to decrease this penalization was the use of ordinary least square weights to each modality when modalities were combined (igure 5, ensemble measures) so that modalities with less contribution would have less impact in the model. We agree that this is a challenging aspect of machine learning approaches in general.

The dynamics measure is complicated. If it is a distance metric, then surely shorter connections are more likely to be affected and/or co-occur together vs. distant connections? Based on Fig. 9D I am not sure how the data speak to this description of finding direct and indirect connections. The metric tells you about the shortest length between nodes, so if there are indirect connections that are not in the node space, then these will be missed in the current analysis.

Response: We appreciate the reviewer's comments. The key point related to dynamics is that it can provide information about indirect information between a pair of nodes, even if they are not directly connected. Consider nodes A and B. CSLM can provide information about the strength of connectivity between A and B only if there are edges in the node space (i.e., connections linking A and B). However, if the shortest oath between A and B is not a direct connection, but rather a long path through other nodes, CSLM is not able to measure this connectivity, but CDLSM is. A more detailed treatment on the subject can be seen in the original description of the approach (Misic et al., 2015), but we have attempted to better explain this point in the text accordingly (Page 20).

Minor:

The hyphens in line 10 of the abstract were difficult to parse.

Response: We have now corrected the hyphens in the abstract.

References

Aerts H, Fias W, Caeyenberghs K, Marinazzo D (2016) Brain networks under attack: robustness properties and the impact of lesions. Brain 139:3063-3083.

Bonilha L, Gleichgerrcht E, Nesland T, Rorden C, Fridriksson J (2016) Success of Anomia Treatment in Aphasia Is Associated With Preserved Architecture of Global and Left Temporal Lobe Structural Networks. Neurorehabilitation and neural repair 30:266-279.

Bonilha L, Nesland T, Martz GU, Joseph JE, Spampinato MV, Edwards JC, Tabesh A (2012) Medial temporal lobe epilepsy is associated with neuronal fibre loss and paradoxical increase in structural connectivity of limbic structures. J Neurol Neurosurg Psychiatry 83:903-909.

Brett M, Leff AP, Rorden C, Ashburner J (2001) Spatial normalization of brain images with focal lesions using cost function masking. Neuroimage 14:486-500.

Crofts JJ, Higham DJ, Bosnell R, Jbabdi S, Matthews PM, Behrens TE, Johansen-Berg H (2011) Network analysis detects changes in the contralesional hemisphere following stroke. Neuroimage 54:161-169.

Gleichgerrcht E, Kocher M, Nesland T, Rorden C, Fridriksson J, Bonilha L (2015) Preservation of structural brain network hubs is associated with less severe post-stroke aphasia. Restor Neurol Neurosci 34:19-28.

Misic B, Betzel RF, Nematzadeh A, Goni J, Griffa A, Hagmann P, Flammini A, Ahn YY, Sporns O (2015) Cooperative and Competitive Spreading Dynamics on the Human Connectome. Neuron 86:1518-1529.

Nachev P, Coulthard E, Jager HR, Kennard C, Husain M (2008) Enantiomorphic normalization of focally lesioned brains. Neuroimage 39:1215-1226.

Schmitt O, Badurek S, Liu W, Wang Y, Rabiller G, Kanoke A, Eipert P, Liu J (2017) Prediction of regional functional impairment following experimental stroke via connectome analysis. Sci Rep 7:46316.

Yourganov G, Fridriksson J, Rorden C, Gleichgerrcht E, Bonilha L (2016) Multivariate Connectome-Based Symptom Mapping in Post-Stroke Patients: Networks Supporting Language and Speech. J Neurosci 36:6668-6679.
